# Invasive occipital nerve stimulation for refractory chronic cluster headache: what evolution at long-term? Strengths and weaknesses of the method

**DOI:** 10.1186/s10194-016-0598-9

**Published:** 2016-02-16

**Authors:** Delphine Magis, Pascale Gérard, Jean Schoenen

**Affiliations:** Headache Research Unit, University Department of Neurology CHR, Boulevard du 12ème de Ligne 1, 4000 Liège, Belgium

**Keywords:** Cluster headache, Occipital nerve, Neurostimulation, Refractory, Adverse events

## Abstract

**Background:**

Invasive Occipital Nerve Stimulation (iONS) is a costly technique which appears effective in drug-refractory chronic cluster headache (drCCH) management. Available data on long-term effectiveness and safety of iONS in this indication are scarce, though they could be useful to neurologists and patients in daily practice. The purpose of this short report is to discuss the very long-term outcome of a drCCH cohort, including adverse events.

**Findings:**

Previously, favourable results were obtained with iONS in 15 drCCH patients: 80 % were significantly improved and 60 % were pain free. We report here the very long-term follow-up (up to nine years) of 10 patients belonging to this cohort. Meanwhile 5 patients had to be definitively explanted because of device infection (3) or paresthesia intolerance (2). Four patients (40 %) evolved to an episodic form of CH. Six remained chronic but their attack frequency was decreased by 70 % on average. Intake of preventive drugs is still necessary in 80 % of patients. All patients needed at least one battery replacement.

**Conclusions:**

Up to nine years after implantation, iONS is still effective in most patients with drCCH. Concomitant preventive drugs remain often necessary. Forty percent of patients reverse to episodic CH, possibly by natural history. iONS is not a benign procedure but device-related complications appear similar to those reported with other invasive neurostimulators.

## Introduction

Cluster headache (CH), especially its chronic form (see [1] for definition), is among the most disabling primary headaches. A small percentage of chronic cluster headache patients (CCH) do not respond to or do not tolerate existing preventive drugs and are considered as drug-resistant (drCCH, [2]). In the last decades various non-pharmacological therapeutic strategies have been applied to relieve these patients, among them invasive Occipital Nerve Stimulation (iONS, [3–8]) that provided middle-term results similar to those of the more invasive and risky hypothalamic deep brain stimulation (hDBS, [9–11]). We published previously a prospective trial of iONS involving 15 drCCH patients [5]. One patient had an immediate device infection and could not be evaluated. After 36 months on average, 11 of the 14 remaining patients (~80 %) had an improvement of at least 90 % in attack frequency, whereas 60 % became pain-free for long time periods. Two patients did not respond or described mild improvement. Up to now, no sham-controlled study of iONS is available in drCCH, but a large trial is ongoing [12].

Recently, Leone et al. [11] published the very long-term outcome (median 8.7 years) of 17 drCCH patients treated with hDBS, and found out that 35 % were still almost pain-free (i.e. less than one attack every three months) whereas another 35 % reversed to an episodic cluster pattern. Unfortunately such data are not available for drCCH patients treated with iONS.

Along the same line, we thus aimed to share some relevant information about the long-term clinical usefulness and especially the risks of this costly procedure, for both neurologists and patients, based on our experience of nearly nine years.

## Summary of methods

**Fig. 1:**
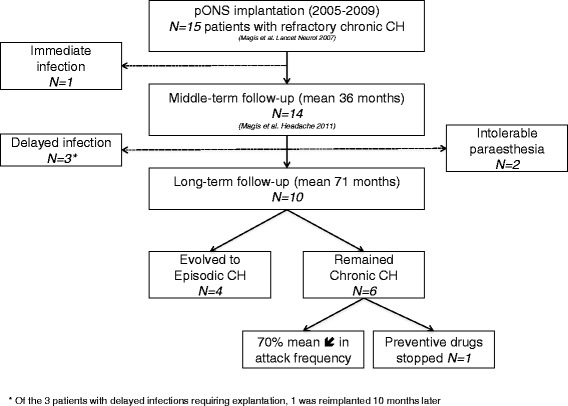
Flowchart of the long-term follow-up. CH = cluster headache

The complete report of the methods and surgical procedure were described elsewhere [3, 5]. Our cohort initially included 15 drCCH patients with side-locked attacks from the start (Fig. 1, see flowchart, one woman, average age at implantation 47.6 ± 9.6 years, duration of the chronic phase 7 ± 4.2 years). In six of them, cluster headache had been chronic from the onset. All subjects gave written informed consent and the study was approved by the Local Ethics Committee, CHR Citadelle, Liège, Belgium. iONS (2005–2009) was performed only on the headache side, using a paddle-style stimulating lead with 4 distal electrodes (Medtronic 3587A Resume II®; Medtronic, Minneapolis, MN, USA) [3]. We used either Medtronic Itrel 3® or Medtronic Synergy® stimulators, and six patients received subsequently a rechargeable Medtronic Restore® when their first battery was empty. The stimulation parameters were adjusted to produce paraesthesia over the greater occipital nerve (GON) territory, covering the largest area of the C2 dermatome.

## Findings

The outcome of the 15 drCCH patients up to 8.6 years after implantation is summarized in Table 1. Five out of 15 patients had their stimulator removed (33 %). Two patients were explanted because they did not tolerate the paraesthesia (at 4 and 35 months, 14 %), although one of them was improved and evolved to an episodic CH. This patient had been chronic from the onset, but remained episodic after iONS removal. Besides the immediate infection in one patient (see Introduction), three more patients were subsequently explanted because of a delayed infection (at 24, 38 and 53 months, 21 %, total rate of infections 27 %). In one of them the attack frequency increased dramatically afterwards, and he was thus reimplanted 10 months later.

**Table 1:** Outcome of the 15 drCCH patients treated with iONS. Patients in grey were explanted due to paraesthesia intolerance or infection

Patients	Age	CH natural history	CCH duration (years)	Time under ONS (months)	Attacks/day before ONS (mean)	Attacks/day at last follow-up (mean) (Magis Headache 2011)	Attacks/day at very long-term follow-up (mean)	% change in attack frequency	Preventive therapy at time of implantation	Preventive therapy at follow-up	Technical problems	iONS	Satisfaction
1	50	E	9	4	0.29	N/A	N/A	N/A	Verapamil	N/A	Unbearable paresthesias: explanted after 4 months iONS	N/A	Not satisfied because of paresthesias
2	53	E	3	103	4.7	0.43	0.33	−93.00 %	Verapamil Melatonine	Lithium carbonate Verapamil	Empty battery: ×3	ON	Satisfied
3	51	E	7	102	3.84	0	0.1	−97.40 %	Lithium carbonate Verapamil	None	Empty battery: ×4 Lead migration: ×1	ON	Very satisfied
4	37	E	4	53	1.16	0.1	0.33	−71.55 %	Lithium carbonate Verapamil	Lithium carbonate Verapamil	Empty battery: ×2 Delayed infection: explanted	N/A	Very satisfied then explanted
5	57	E	4	38	0.16	0	N/A	N/A	Verapamil	N/A	Delayed infection: explanted	N/A	Not available
6	34	C	6	95	0.16	0	Episodic	Episodic	Lithium carbonate Verapamil	Verapamil Lithium carbonate Topiramate	Empty battery: reluctant to replacement	OFF	Satisfied
7	63	E	5	95	1.00	0	0.17	−83.00 %	Methysergide Lithium carbonate	Lithium carbonate	Empty battery: ×3	ON	Satisfied
8	51	E	3	83	4.00	0	1	−75.00 %	Verapamil Methylprednisolone	None	Empty battery: ×1	OFF	Not satisfied
9	53	C	29	35	1.5	0.16	Episodic	Episodic	Verapamil Lithium carbonate Methysergide	During bouts: GON injection, verapamil, lithium carbonate	Unbearable paresthasias: explanted	N/A	Not satisfied because of paresthesias
10	33	E	5	68	2.00	0	Episodic	Episodic	Verapamil	Verapamil Gabapentine	Empty battery: ×1	ON	Satisfied
11	46	C	2	64	0.57	0.5	0.54	−5.26 %	Verapamil Lithium carbonate Gabapentine Escitalopram	Verapamil Gabapentine	Delayed device infection: explanted and reimplanted	ON	Moderately satisfied
12	34	E	8	na	na	na	N/A	N/A	Methylprednisolone	N/A	Immediate device infection: explanted	N/A	N/A
13	67	C	5	58	3.5	0	1	−71.00 %	Lithium carbonate Verapamil	Lithium carbonate Verapamil	Empty battery: ×1	OFF	Not satisfied
14	55	C	2	57	5.5	0	Episodic	Episodic	Methylprednisolone Methysergide Clomipramine	GON injection Verapamil	Empty battery: ×1	OFF	Not satisfied
15	30	C	14	54	3.00	0	Episodic	Episodic	Methysergide Topiramate Verapamil	GON injection Verapamil Lithium carbonate	Empty battery: ×1 Lead externalization	ON	Not satisfied

*E* evolved from an episodic to a chronic pattern, *C* chronic since the onset, *N/A* not applicable

The remaining 10 patients have a mean follow-up of 71 months (Table 1, range 54–103). CH attacks recurred in all patients who were pain-free at the previous middle-term follow-up. In four patients (40 %), attacks relapsed following an episodic pattern. The bouts responded to standard preventive therapies (suboccipital steroid infiltration, verapamil …). The other six patients (60 %) became chronic again [1] with an mean attack frequency ranging from 3 to 30 per month, which represents a reduction of 70.8 % on average, compared to baseline (Table 1). However, 8/10 patients (80 %) still need preventive medications but only 5/10 (50 %) are still stimulated (two are episodic and three chronic). Their main explanation to discontinue iONS was their improvement which persisted despite an interruption of the stimulation due to various reasons (cancer, empty battery…). Overall, compared to baseline period, 9/10 patients have at least a 50 % decrease of attack frequency. Six are satisfied with the treatment. The need for repeated surgery is the main reason for patient’s dissatisfaction. Hence, patients stimulated at long-term had to undergo at least one additional surgery for battery replacement (up to four/patient). Two patients also needed surgery for lead migration (2/10: 20 %). Some transient attack side-shifts (a single bout or isolated attacks) had been observed previously in nearly 30 % of patients, but were not reported during the subsequent follow-up.

## Discussion

Our data confirms that iONS is able to provide a long-lasting relief in a majority of drCCH patients nearly 10 years after implantation.

All patients stimulated at long-term underwent at least one additional surgery for battery replacement, but the stimulators implanted initially had a limited lifetime and were expected to deplete after a time period depending on the stimulation intensity. Thus, a rechargeable device was placed subsequently to ensure a longer-lasting stimulation. Fifty-three percent of patients developed iONS-related complications, like immediate or delayed infections which finally required explantation of nearly 30 % of patients. Besides the small size of our sample which could have biased the results, this high number of adverse events can be explained by several factors. First, the duration of our follow-up period is exceptionally long. The cumulated rate of adverse events probably increased with time and surgeries (especially repeated device replacements). Second, few similar safety data are available in the literature. A high complication rate was reported in chronic migraine patients treated with iONS [13]. In a cohort of 157 patients, after 1 year follow-up only, the authors recorded 183 device/procedure-related adverse events, among which 8.6 % required hospitalisation. Overall 32.5 % of patients needed additional surgery; 16.6 % had lead migration, 6.4 % infection, 4.5 % skin erosion and 18 % local pain or numbness. Besides the rare but possibly fatal risk of intracerebral haemorrhage, hypothalamic deep brain stimulation (hDBS), has similar long-term complications such as infections (5/18, 1 immediate, 28 %), electrode migration (2/17, 12 %), or need for battery replacement (6/17, 35 %) [11]. Larger long-term data are available for invasive vagus nerve stimulation in intractable epilepsy, and authors report side effects in 50 % of patients, with surgical complications in 21 % [14].

Our clinical data support that iONS is no more than a symptomatic therapy, as suggested before by other clinical [3] and neuroradiological [15] observations. iONS likely induces slow neuroplastic changes within non-specific pain-control systems [3], which explains its beneficial effects in various headache types. The evolution of our patients was characterized by a sustained pain relief, even in some patients who had discontinued iONS (see Findings section). Forty patients became episodic and 60 % stayed chronic, but many still needed a concomitant drug prophylaxis. With hDBS, after a similar follow-up time, 35 % of drCCH patients remained ‘almost’ pain-free [11], but the chronic phase duration, which could mirror the disease severity, was on average twice longer in our population (seven years vs. three years for hDBS). However, comparing the outcomes of both techniques is challenging due to the small size of the series [11]. Interestingly, three patients who had been chronic from CH onset developed an episodic form after an initial pain-free period under iONS. A similar evolution from a pain-free state to an episodic form of CH has been described under hDBS in 35 % of patients (6/17) [11], however they were still stimulated; whereas the stimulator was turned off in the half of our population. Leone et al. suggested that hDBS might have changed the course of the illness by acting on circuits involved in disease chronification [11]. We have reported before that iONS applied during several months modulated central areas involved in non-specific pain control but did not modify the hypothalamic hypermetabolism found in CH [15]. It is also known that about 32 % of “primary” CCH patients can spontaneously evolve to a “secondary” episodic type [16]. Thus, the emergence of an episodic pattern after iONS could either be due to the natural course of the disease, or be favoured by iONS.

In this trial, iONS had been performed unilaterally (headache side), in patients with strictly side-locked attacks. We previously observed a headache side shift in 4 patients [3, 5], but the latter was transient and fortunately did not recur at long-term. To avoid a possible attack side shift associated with chronification a bilateral stimulation is now proposed in drCCH patients.

## Conclusion

Available open studies using iONS as add-on therapy have provided encouraging results in drCCH, and iONS is now recommended before considering the more risky hDBS [17]. The very long-term evolution of patients treated with this technique is unknown.though, but this missing information is important for both neurologists and patients when considering invasive neurostimulation. Based on a 9-year experience, our data show that iONS remains very effective in patients who had initially benefitted from the procedure. That iONS did not prevent any relapse confirms its purely symptomatic effect on pain-controlling centres. Over time 40 % of patients reversed to an episodic pattern of CH, perhaps by natural history. This proportion is actually similar to the rate found in medically-treated CCH patients [15], but the cohort studied here involved a subset of the most severely ill drCCH sufferers, some of them being in chronic phase for several dozens of years. The treatment could thus have modified the course of the disease through a slow neuromodulation phenomenon.

Like other invasive neurostimulation techniques, iONS is not harmless and its use in drCCH patients should be considered carefully. Adverse events occurred in about 50 % of the subjects over time, but does not seem to deviate significantly from cumulated rates described with other techniques at very long-term (hDBS and VNS). The refinement of surgical techniques and devices will probably reduce the hardware-related complications. It is also strongly suggested to refer patients to trained surgeons who are familiar with iONS placement [13]. Patients must be aware that additional surgeries may be needed to replace empty batteries (also rechargeable batteries have a limited lifetime).

At present, because less risky than hDBS, iONS is recommended to the most disabled drCCH patients when invasive neurostimulation is considered. If available, non-invasive neurostimulation devices like vagus nerve [18] or transcranial direct current stimulators should be tried before performing invasive procedures (see European Headache Federation Statement [17]).

## Abbreviations

CH: cluster headache
drCCH: drug-resistant chronic cluster headache
GON: great occipital nerve
hDBS: hypothalamic deep brain stimulation
iONS: invasive occipital nerve stimulation
